# New World Primates and Their Human Counterparts Share Diseases That Abound with CEACAM and Other Effector Molecules

**DOI:** 10.3390/life15030481

**Published:** 2025-03-17

**Authors:** Martin Tobi, Daniel Ezekwudo, Benita McVicker, Harvinder Talwar, Laura Kresty, Elizabeth Curran, Ronald Veazey, Peter J. Didier, James Hatfield, Mike Lawson, Sonia M. Najjar

**Affiliations:** 1 Department of Research and Development, Veterans Health Administration, Detroit, MI 48201, USA; james.hatfield@sbcglobal.net; 2 Department of Oncology, Corewell Health William Beaumont University Hospital, Royal Oak, MI 48773, USA; 3 Department of Internal Medicine, University of Nebraska Medical Center, Omaha, NE 68005, USA; bmcvicker@unmc.edu; 4 Department of Research and Development, Wayne State University School of Medicine and Veterans Health Administration, Detroit, MI 48201, USA; ar8673@wayne.edu; 5 Department of Thoracic Surgery, University of Michigan, Ann Arbor, MI 48109, USA; lkresty@umich.edu; 6 Yerkes Primate Center, Emory University, Atlanta, GA 30322, USA; ecurram@emory.edu; 7 Tulane University Primate Center, New Orleans, LA 70433, USA; rveasy@tulane.edu (R.V.); pjdidier@tulane.edu (P.J.D.); 8 Department of Internal Medicine, University of California at Sacramento/Davis, Sacramento, CA 95817, USA; aus.mlawson@att.net; 9 Department of Biomedical Sciences, Ohio University, Athens, OH 45701, USA; ssmnajjar@ohiostate.edu

**Keywords:** primates, monkeys, cotton top tamarins, common marmosets, adhesion molecules, carcinoembryonic antigen

## Abstract

**Background:** Herein, we review the Cotton Top Tamarin (CTT), *Saguinus oedipus*, a unique spontaneous model for colorectal cancer (CRC). Despite its predisposition to inflammatory bowel disease (IBD) and frequent development of CRC, the CTT is adept at avoiding colorectal metastasis in the liver. In contrast, the common marmoset (CM), *Callithrix jacchus*, is a natural negative control, in that it also contracts IBD, but usually not CRC. We review our findings in these New World monkeys in terms of the expression of CEACAM adhesion models and their related molecules to contrast them with human disease. **Methods:** Specimens were collected from aforementioned monkey colorectal and other tissues, colonic washings, serum for analysis of tissue extraction, and colonic washings via ELISA, using a battery of antibodies. Fixed tissues were analyzed using immunohistochemistry and CEACAMs were extracted via Western blotting. Serum CEA levels were analyzed using ELISA, and DNA was extracted via a Bigblast genomics sequencing kit. **Results:** Serum CEA was significantly elevated in CTTs, and one-third of them die from CRC. Unlike others, we were unable to stain for CEA in tissues. The sialylated carbohydrate antigen recognized by monoclonal antibody (MAb) SPAN-1 does stain in 16.7% of CTT tissues, but the anti-aminoproteoglycan MAb, CaCo.3/61, stained 93.3% (OR70·00[CI6.5–754.5] *p* < 0.0001). The common CEA kits from Abbott and Roche were non-conclusive for CEA. We later adopted a CEA AIA-PACK from Tosoh Medics, which identified a 50 Kda band via Western blotting in humans and CTTs. The CEA levels were higher using the CEA AIA-PACK than the Pharmatrope kit (932 ± 690 versus 432 ± 407 ng/mL (*p* < 0.05)) in human patient colonic effluent, not statistically significant (NSS) for CTT extracts or effluent (733 ± 325 and 739 ± 401 ng/mL, respectively). It was suggested that the smaller CTT CEA moiety might lack components that facilitate the spread of liver metastasis. Later, using more specific CEA assays and increased numbers of specimens, we were able to show higher CEA serum expression in CTTs than in CMs (632.1 ± 306.1 vs. 81.6 ± 183.6, *p* < 0.005), with similar differences in the serum samples. Western blotting with the anti-CEA T84.66 MAb showed bands above 100 KDa in CTTs. The profiles in CTTs were similar to human patients with inflammatory bowel disease. We established that the CEA anchorage to the cell was a GPI-linkage, advantageous for the inhibition of differentiation and anoikis. With further CEA DNA analysis, we were able to determine at least five different mechanisms that may inhibit liver metastasis, mostly related to CEA, but later expanded this to seven, and increased the relationships to CEACAM1 and other related molecules. Recently, we obtained CTT liver mRNA transcriptomes that implicated several pathways of interest. **Conclusions:** With efforts spanning over three decades, we were able to characterize CEA and other changes that allow us to better understand the CTT phenomenon of liver metastasis inhibition. We are in the process of characterizing the CTT liver mRNA transcriptome to compare it with that of the common marmoset. Currently, liver CTT gene expression patterns suggest that ribosomes, lipoproteins, and antioxidant defense are related to differences between CTTs and CMs.

## 1. Introduction

Animal models of human disease have been extremely useful for understanding cancer pathways and effective interventions, but few can match quintessential spontaneous inflammatory bowel disease (IBD) as the prelude to subsequent colorectal cancers (CRCs) contracted by the Cotton Top Tamarin (CTT), *Saguinus oedipus* [1], a resident of northwestern Columbia. While one-third of CTTs succumb to CRC, less than two percent have liver metastasis [2], and the underlying mechanisms of avoidance are of acute relevance to humans with cancer, where liver metastasis is a harbinger of death in 70% of cancer victims, and 20–35% have liver spread at the time that the primary cancer is diagnosed [3]. Biomarkers are currently available for the early diagnosis of CRC in humans, but CTT cancer biomarkers in lower order primates have only recently been described and their genomes registered [4,5]. Some researchers point to stress and the microbiome as a contributor to the development of CRC [6,7]. The CTT is also subject to the development of lymphomata and known to be susceptible to infections such as Epstein–Barr virus and Coronavirus [8,9,10], suggesting an element of immune deficiency. Its Brazilian cousin, the common marmoset (CM), *Callithrix jacchus*, also contracts IBD, but usually not CRC, providing a convenient control. The carcinoembryonic antigen was first discovered in humans in 1965 [11] and almost a quarter of a century later in apes and chimpanzees [12]. Seven years thereafter, a mouse carcinoembryonic antigen (CEA) analog was discovered [13], which ushered in burgeoning interest in this model. This was the era of monoclonal antibodies, which provided the essential tools to demystify markers on CTT lymphatic cells [14,15]. This paper will follow attempts to characterize and detail adhesion molecules and antigens in broad detail and attempt to define their role in the avoidance of liver metastasis, possibly extending these observations to humans.

## 2. Methods

The animal tissues were initially procured from two main sources, the Marmoset Research Center, Oak Ridge, Tennessee, and the New England Regional Primate Center, Boston, MA, USA. When the latter center closed, the tissues were transferred to the Tulane National Primate Research Center, Covington, Louisiana, USA. Tissues were only obtained from deceased or terminally ill euthanized animals, since the CTT has been on the endangered list since 1973. Where comparisons with humans with IBD were made, colon washings and tissue biopsies were obtained from patients of the Kaiser Permanente Medical Center, Sacramento, CA, USA, and later from a longitudinal study conducted at the Detroit Veterans Administration Medical Center, Detroit, Michigan, USA, with both centers subject to institutional board review (IRB). Briefly, extracts were prepared using Dounce, homogenized with 1 gm/10 mL of saline and magnesium chloride, and centrifuged at 1000× *g* in Sorvall RC-5B refrigerated centrifuge. The supernatant was aspirated and subjected to a high speed of 10,000× *g*, the supernatants represent the membrane enriched extract, and the protein content was determined as described below.

Common methods for analysis were enzyme-linked immunosorbent assay (ELISA), Western blotting (WB), and immunohistochemistry (IHC). The ELISA utilized 96-well microtiter plates (Nunc, Copenhagen, Denmark), in which samples with identical protein content (5 µg/well) were determined via a Lowry colorimetric assay. A Vector Laboratories kit with the primary monoclonal antibodies (shown in Table 1) or control antibody (UPC10, Sigma-Aldrich, St Louis, Mo, USA) was used to determine the background reaction, and secondary antibodies to either IgG or IgM linked to alkaline phosphatase. There were 3 consecutive washes between successive applications of 5% Tween-20 in phosphate-buffered saline (PBS). The final step was to add p-p-nitrophenyl-phosphate substrate (Millipore-Sigma, St. Louis, Mo, USA), which gives a yellow color and was measured using a spectrophotometer (Thermo-Fisher, Waltham, MA, USA) at 405 nm for 30 mins. The results were reported as optical density (OD) minus background/5 µg per well.

Western blotting was performed on a BioRad apparatus (Hercules, CA, USA), loading 10 µg of protein per well of extract. Then, 9.6% SDS-polyacrylamide gels (Millipore Sigma, St. Louis, MO, USA) were loaded at 10 ug/lane on SDS-gels and electrophoresed according to the manufacturer’s directions. The gel proteins were transferred to a milk powder-blocked PVDF membrane (Kirkegaard and Perry, Gaithersburg, MD, USA), which was then reacted with the primary and secondary antibodies as described above, and then with a number of substrates for alkaline phosphate or peroxide, depending on the linker attached to the secondary antibody. A lane for the molecular weight ladder (Thermo-Fisher Waltham, MA, USA) was used to determine the relative mobility of the protein in question, and its picture was documented.

Immunohistochemistry was performed on paraffin-embedded tissue fragments, properly oriented, and cut with a microtome to a thickness of 5 µm. After deparaffination and hydration steps, the slides were reacted with selected MAbs, and the Vector kits (Vector Labs., Newark, CA, USA) were used to develop a color with the available substrate, such as alpha-amino benzidine to produce a black color with added zinc chloride. The slides were then cover slipped with a clear adhesive and examined at various magnifications, and photographic documentation was duly noted. The grading system for intensity that we used is given as follows: 0—no stain; 0.5—equivocal stain; 1+—definite but faint, 2+—medium intensity; 3+—strong intensity; and 4+—very strong intensity.

### 2.1. Study Outcome and Data Collection

Selected monoclonal antibodies that are reactant with biomarker outcomes were obtained from a number of sources and are tubulated in Table 1 above. They were used in enzyme-linked immunoassays (ELISA), immunohistochemistry, and Western blotting experiments.

### 2.2. Hierarchical Clustering and Clustering Process

Often, particularly when different primate species are compared, teleogenetic trees need to be considered and organized accordingly. Since CRC has been observed in several species, these differences need to be compared. It is safe to say that cancer is not a common disease in most non-human primate species. The CTT and, less frequently, the closely related CM, have a higher incidence of CRC and small bowel cancers, respectively, than other non-human primates (NHP), and other tumor types are common for these 2 species. The genomics that we performed with the BigBlast Dye Terminator methodology [2] have shown significant differences, but in the CEA Ig pivot region (hinge), where the PELPK pentapeptide is located, the differences appear to be muted.

### 2.3. Ethical Approval

As noted above, monkey tissues were obtained from registered biorepositories at one of the three Primate Research (New England, Yerkes, and Tulane) Centers. Earlier samples were obtained from the Marmoset Colony at Oak Ridge (MARCOR), Tennessee. Human samples for comparison were obtained from patients enrolled in the NIPCON study, approved by the Wayne State University School of Medicine IRB, Wayne State University Human Investigation committees, #070700MP4F, and all patients gave informed consent.

### 2.4. Statistical Analysis

The statistical analysis was performed using a computer statistical package (instat, version 2, Graphpad Software Inc., San Diego, CA, USA). The normality of values was tested using the online Kolmogorov–Smirnov calculator, https://www.socscistatistics.com/tests/kolmogorov/, accessed on 5 December 2023. The ordinal data were analyzed using Student’s *t*-test or the Mann–Whitney test for non-normality values. The non-ordinal data were analyzed using the Chi-square test or the Odds Ratio, and the confidence interval value proportions were presented as percentages in graphs, with the *p* values depicted. Linear correlations were analyzed using the least-squares method, and the r coefficient values were depicted on the relevant graph. The probability values were regarded as significant at the <0.05 level.

## 3. Results

### 3.1. Analysis 1: Immunohistochemistry

Tabulated below in Table 2, is the composite of results with the citation in which the data were originally reported.

**Figure 1:**
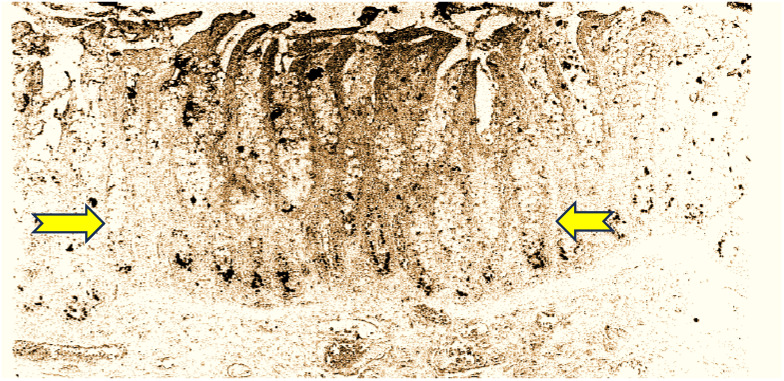
CaCo 3/61 staining of CTT colonic crypts shows that the bottoms of the crypts are intensely stained (between yellow arrows), similarly to rat crypts, as has been previously described. The cellular distribution is focally cytoplasmic and the intensity is 3–4+, as described in the methods section.

The anti-CEA used in this series was the highly sensitive and specific MAb T84.66. It has been consistently used for human CEA testing, mainly in human sera, and is the basis for the Roche CEA immunoassay, and superior when compared to the CEA RIAKIT. However, the authors did disclose that they “slightly modified” the Roche kit, and found the first to have a greater affinity for tissue CEA than blood CEA [18]. This observation was tested 15 years later in a Callitrichid application, leading us to reopen the discussion on the CTT as a relevant model [19]. It should not come as a surprise that anti-CEA antibodies have a vast spectrum of specificity, with some “questionable” antibodies being some of the first to recognize the Callitrichid CEA moiety. SPAN-1 is a sialylated high-molecular-weight mucin and is related to the epitope related to CA19-9 of the IgG1 isotype. This antibody and other related epitopes were also used in IHC in the CTT, such as MAb 29-1, an IgM isotope which reacts with a lacto-N; fucopentaose III, an Le^x^ antigen; and E5-6, detecting an Le^b^ antigen. Only the latter showed staining in 50% of tumor cells [20]. Other antibodies were also used, but the results were not fully conclusive. CaCo 3/61 readily stains CTT colon tissues and rat jejunal cells, and in humans, seems specific for adenocarcinoma, but seems to bind CTT tissues universally. The strongest 4+ staining occurred in the lumen of the crypts, suggesting a semi-permanent location (see Figure 1).

### 3.2. Tissue Extract and Colonic Washing ELISA

Tabulated below in Table 3, we summarize the labeling of tissue extracts and colonic washings.

We used standard ferritin 215 ng/mL as the plasma mean value, as determined by AMSBIO, Cambridge MA, USA, from healthy African green monkeys and Cynomolgus macaques (Yusheng Qin—personal communication). While CEA kits are quantitative, the working dilutions of SPAN-1 and CaCo 3/61 are typically 1:500.

**Figure 2:**
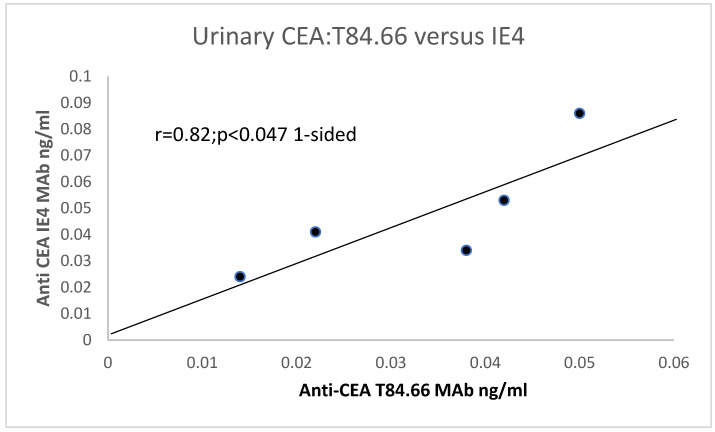
There is a putative positive correlation between T84.66 and another CEA (MAb IE4). Legend to Figure 2: In control human patients, urinary CEA is 0.9 ± 0.77 from an Egyptian study of humans with Bilharzia [22], using the CEA kit, procured through Abbott for both serum and urine for direct comparison. There is a direct correlation. An inverse relationship between serum and urinary CEA can be seen in Figure 3.

Urinary CEA was also measured using T84.66 and another highly specific IE4 antibody (see Figure 2), and a one-sided significant correlation was found. This suggests that urinary CEA may be useful in detecting urinary tract tumors and even bowel tumors. Using the specific T84.66 MAb, in CTT urine samples, the mean ± sd was 0.033 ± 0.015; *p* < 0.039 (please see below). Not many higher molecular urinary proteins are found, and those proteins, albumin and transferrin, usually manifest in disease states [21]. CTT CEA levels in extracted colonic mucosae are clearly much higher in colon extracts than in urine (*p* < 0.0078).

We could also contrast colon extracts from CTT with and without cancer and CM as controls in Table 4.

**Figure 4:**
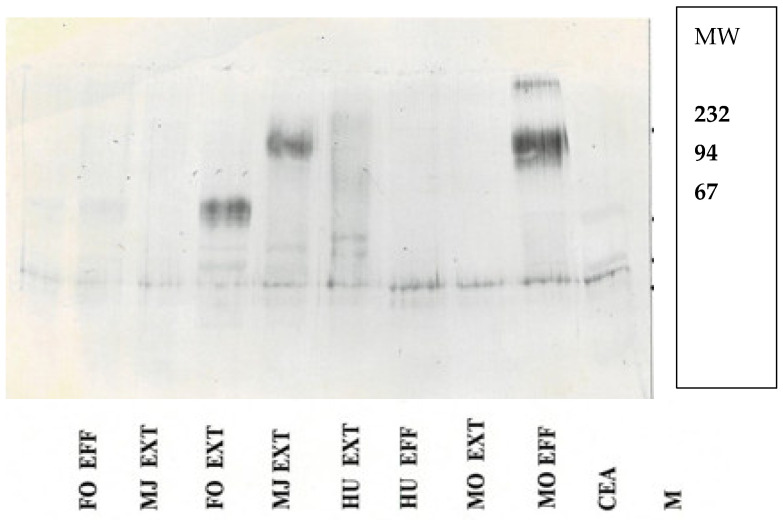
A Western blot shows different Mr between Callitrichid colonic effluents and extracts. CMs have ~90 kDa CEA band, as opposed to the 220 kDA of human CEA. Legend to Figure 4: A complete Western blot of CEA molecular weights indicated on the right and the animal source at the bottom. Abbreviations are FO—female Oedipus; MJ—male Jacchus; HU—human; MO—male Oedipus; EFF—effluent sample; EXT—membrane-enriched extract; CEA—carcinoembryonic antigen; and M—reference molecular weights. CEA is ~180,000, identical to the human extract, and ~90 kDa from the MJ extract.

Western blot of tissue extracts and colonic washings are shown Figure 4.

### 3.3. CCT and Avoidance of Liver Metastasis

This aspect of the CTT is probably the most compelling to which we could apply the following adage: “The Lord Invents the Cure before the Disease” [23]. At the outset, the pathogenesis of colon cancer in the CTT is still contentious. Some lay the blame for the high rate of CRC in the CTT on its immune system, which confers upon the CTT “model status” a number of diseases, including CRC [3]. Others contend that environmental stress and stool factors are likely contributors to the CTT’s high CRC incidence [6]. This was also the view of one of the early human IBD researchers (Personal communication—Dr. Moshe B. Goldgraber). The high incidence and mortality of CTT CRC is the reality, and CEA is thought to play a major role in the tumorigenesis of CRC [24]. At the other end of the CEA molecule is the interface with the cell membrane, and the type of molecular attachment may also confer a susceptibility to cancerogenesis [25,26]. Using the Big Dye Terminator^TM^ kit and the ABIPRISM 377^®^ system (Applied Biosystems, Foster City, CA, USA), we sequenced the hinge region of CEA to identify changes in the PELPK pentapeptide in 19 Callitrichids, of which 12 were CTT and 7 were CM [2]. More than half of the CTTs showed changes (58.3%), and only two of the CMs (28.6%). We also stained for the CEA receptors (CEAR) in the liver, and because CEACAM1 (biliary glycoprotein) is a downstream VEGF effector, we included it in our staining repertoire and showed that there was little glycosylation in Callitrichid CEA [27]. Later, we added fibulin-5 stain, which is anti-neoplastic, to bring the number of putative liver metastasis avoidance mechanisms to five [3], Table 5 lists all the known mechanisma by which the CTT avoids metastasis.

**Figure 5:**
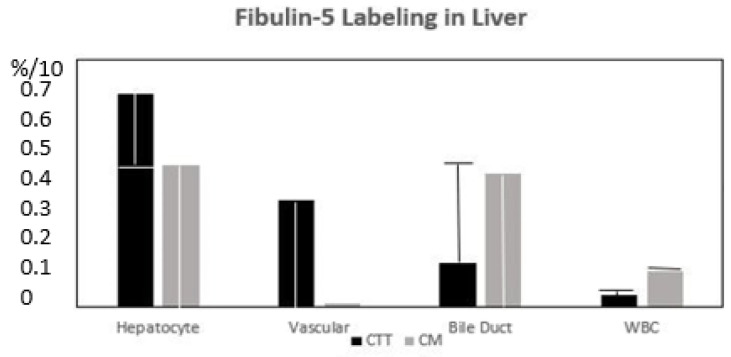
The distribution of staining in locations of the liver with no significant differences.

PELPK mutations in humans were associated with malignant ascites, but no detectable liver metastasis [28]. This is somewhat analogous to the CTT. The lack of BGP (CEACAM1), a downstream effector of VEGF, probably inhibits the seeding of liver metastasis. In humans with liver metastasis, BGP translocation occurs from an inactive biliary canaliculus structural protein to the hepatocyte, where it can be active in the VEGF cascade. Increased CEA glycosylation has been associated with increased liver metastasis in humans, while the inhibition of N-glycosylation slows metastasis [29], and reduced CEA receptor expression would be expected to associated with less metastatic spread. Phosphorylated pp38γ is not found in the CTT’s liver, which would not be expected to promote metastasis, but interestingly, pp38γ is found in the ileum of the CM, which is susceptible to adenocarcinoma. Fibulin-5 has direct anti-metastatic activity [3] and is found in the hepatocytes surrounding the central vein (see Figure 5 below).

After concluding that the Callitrichid liver is an organ inhospitable to metastases, we further examined the role of inflammation in CTTs and CMs with regard to ICAMs. While we did not find CEACAM1 in the liver itself, it was clear that it was expressed in the tamarin’s gallbladder. We then compared CEAMCAM-1 with the expression of other antigenic epitopes in the animal tissue extracts. We found that other anti-CEA antibodies [30,31] underwent binding that compared well with the level of CEACAM1 (CEA superfamily 53.5 and CEA 46.1 (80–100%)). As anticipated, T84.66 binding was as low as 10%. Other recognized epitopes 8–32% were sialylated FH2 Le^x^, extended KH1 Lewis Y blood group, n-sialyl Tn, and CaCo 3/61 fucosylated aminoproteoglycan. Sialylated FH6 Le^x^ fucoganglioside 6B tied with Adnab-9 at 60%. The positive correlations with CM were CEAMCAM1 and CaCo2 (r = 0.96; *p* < 0.01), but this was not seen with the CTT. Both animal subspecies showed a positive correlation of CEACAM1 with Adnab-9 in tissue extracts (r = 0.521; *p* < 0.047). Surprisingly, T84.66 highly correlated with Adnab-9, but only in CTT extracts (r = 0.91; *p* < 0.0004). Extracts from normal-appearing CTT mucosae only correlated with CEA obtained using the CEA AIA Pack kit correlated with CEACAM1. We compared p38γ between human CRC extracts and CTT. The CTT does not appear to express phosphorylated p38γ, which is important for metastasis in the liver, but the CM does. However, p38γ is not present in the CM colon and is not prone to CRC, despite colitis. Interestingly, the CM ileum does express p38γ, and this bowel location is the most common for adenocarcinoma of the ileum in aging CM [32] (Table 6).

In an attempt to examine which proteins and pathways are activated in the CTT and CM livers, we obtained mRNA transcriptomes from 6 CTT and 6 CM livers. We then generated a map of expressed and non-expressed proteins. Currently, there is a sequenced genome available for the CTT [5], and we could now use the transcriptome sequences to identify the liver expression of proteins possibly relevant to liver metastasis.

**Table 6:** Summary of some of the statistical outcomes of our non-human primate study results, comparative to humans.

Parameter	Humans (n)	Monkeys	Comparison	Statistics	*p* Value
Age (years)	58.8 ± 8.80	9.73 ± 5.31	Both > Middle age	CRC vs. no	<0.007 *
Sex (M/F)	9:1 (10)	37:19 (46)	22:13ctt 15:6 CM	M > F	0.057
Degree IBD/Type	6 active IBD	15:1 active	60 vs. 94%	Borderline	0.055
FERREFF ratio	6867 ± 162,761:9	83,574 ± 136,321	Higher in monkeys	*t*-Test	<0.044 *
CRC vs. no cancer	1:9 (breast)	19 + 17-	or 10.06 [1.15–97.85]	Chi-square	<0.028
Other cancers	6 + 4-	4 + 33- ^	NSS	Chi-square	0.26
P87 Effluent +		2 + 9-	60 vs. 18.2%	Chi-square	0.08

* Age compared CTT with and without cancers; ^ sarcoma mandible; kidney cancer; small bowel cancer; jaw cancer. We used standard ferritin 215 ng/mL plasma as mean value, as determined by AMSBIO, Cambridge MA, USA, from healthy African green monkeys and Cynomologus macaques (Yusheng Qin—personal communication). NSS = not statistically significant; + *p* > 0.05 (OD-background).

Table 7 provides a numerical accounting of the number and type of experiments beformd.

**Table 7:** Brief summary and list of numbers used to derive numbers for individual experiments as a supplement to Table 5. We introduced a table to summarize the specific monkey and human experiments from which we derived data.

Experiment Numbers	Cotton Top Tamarin	Common Marmoset	Human
Western blotting	10	2	2 (for Reference)
Genetic PELPK CEA seq	19	17	1 (for Reference)
Liver immunohistochemistry	6	6	38 (6m, 14ch12c6nl)
Serum CEA	5	5	12
CEA Liver Receptor IHC	6	5	28

n-number PELPK (Proline–Glutamine–Leucine–Proline–Lysine); CEA—carcinoembryonic antigen; seq—sequence; IHC—immunohistochemistry; m—metastasis to liver; ch—chronic hepatitis; c—cirrhotics; and nl—normal.

## 4. Discussion

For more than three decades, we have toiled to understand the mechanisms of disruption of liver metastasis and now believe that we stand before the final gate. While CRC is a leading cause of cancer death and is mediated via the final common pathway of liver metastasis, leading to death in nearly 70% of CRC cases, many other common cancers share this final common pathway [32]. p38γ probably plays a role in liver metastasis, and there are at least two relevant p38γ blockers of this pathway that have been used in clinical studies, and which we included in our last study [32]. p38γ induces *ras*, which may play a role in human cancer and metastasis, but does not appear to be active in CTTs, while the emphasis on non-human primate work appears to be moving in the direction of adopting CMs as an animal model, as they are currently not an endangered species.

The comparison of humans with IBD and cancer with the IBD therapeutic response in CTTs is interesting. The CTT is dependent on the same medicines used in IBD, and we have much to learn from CTTs with respect to cancer and IBD developments. Just after the turn of the last century, we had already noted differences in the repertoire of antigens and were interested to find and document differences in the form of IBD and incipient cancers arising from the IBD risk factor [31]. Particularly of interest, the T84.66 MAbs directed at the N-terminal “hinge” portion of the CEA molecule did not always react, and we suspected that this may be caused by mutations in the recognized epitope [3,29]. This avenue of investigation had been opened by the discovery of the genetic sequence of CEA [29] and homology between other members of the CEA–immunoglobulin gene superfamily [30]. We found quite a range of reactivity to CEA using the CEA AIA kit and smaller variation in CEA size clustering, as revealed by T84.66 MAb [31].

K*ras p21*, EGFR, neuroendocrinal factors (chromogranin-A), and telomerase [33] may be operative in both human and CTT CRC as common gene mutations. This enzyme maintains the length of the TTAGG at the ends of the chromosome, and as the cell ages, telomeres are lost due to this process of replicative senescence [34]. Some cells may escape this process via telomerase reactivation, and their increased survival may lead to genetic mutations and the possibility of irreversible carcinogenesis. Thus, increased telomerase activity may be used as an early biomarker of cancer detection. We therefore investigated these factors using MAbs and the Trapeze assay in the CCT and the human. K-*Ras* p21 was absent in the CTTs, and they do not seem to express the common gene mutations (*KRAS*).

Chromogranin-A did not differentiate between cancer CTT tissue or normal resident neuroendocrine cells. However, Adnab-9 MAb binding (adenoma biomarker 9) and now the likely innate immune system indicator, was higher in normal humans (*p* < 0.05) than IBD washings, and also positive in IBD human extracts. K-*Ras* p21 was elevated in humans with IBD (*p* < 0.05). EGFR was maximal in CTT extracts (*p* < 0.005), known to occur in 80–90% of human CRC [27]. Aminoproteoglycans (APGs) were also maximal in the CTT extracts, and given their widespread distribution in CTT (see Figure 1), this is not surprising. Telomerase is known to be low in human washings or tissue [33], and 86% of our human IBD samples were negative, but in contrast, over half of the CTT washings were positive, supporting their role in the early detection of cancer [24].

In terms of research interest, there were 5187 Pubmed entries since 1930 for CMs compared to 1149 for CTTs since 1965, the date of the outset of largescale exportation to pharmaceutical companies, research facilities, and the like. Only 18% of the above publications center on CTTs, although there will be overlap due to certain articles featuring both CTTs and CMs [10,25,31,32,33,34,35,36,37,38]. The peak for CTTs was in the year 2000, with 43 articles, and the curve conforms to a Gaussian distribution, with only 8 publications for 2024 thus far. If we are able to complete our genome work, we do anticipate an uptick in interest.

For the interested reader, below we provide a description of meetings and workshops to show that it was these goodwill meetings that have advanced the field into this epoch of discovery. At this juncture, the following is the expected trajectory of what we hope to achieve within this year, until the time of the next CEA workshop in Wurzberg, FRG.

If the CTT is the bulwark against distant metastasis, we recognized that only a thorough evaluation and mapping of all extant pathways in the livers of the Callitrichidea clades would need to be completed. A team was put together to further this end. The Tulane and Yerkes Primate Centers kindly offered to provide the necessary livers. Work to purify mRNA from the livers began at the University of Michigan in the Laboratory of the Kinesiology Department. This resulted in the passable quality of the mRNA liver transcriptomes and these were sequenced. The reads that were obtained were subjected to bioinformatics scrutiny, with a number of pathways obtained. The major impediment to obtaining full bioinformatics was relying on genomic sequence data that could be compared and contrasted between the CTTs, CMs, and humans. Genomic data were available for the CM and the human, but not for the CTT, and substantial funds would have been needed to achieve a full genomic CTT sequence. Providence provided us with the full sequence published by Rasmussen et al. in a masterful international collaboration, for which they are deserving full accolades. Funds were generously provided, and we are currently probing the resultant transcriptomes with a spinoff bioinformatics company affiliated with Wayne State University. The authors of this paper wish to make it clear that without participation and international collaboration over many decades, we would not be standing on the threshold of what may very well be a major breakthrough for us primates.

It is therefore perhaps timely to reflect on what we have learned from these animals. The first peak of CTT-related publications was in 1985. The impetus for this effort was the workshop entitled as follows: Is the Marmoset an experimental model for the study of gastrointestinal disease? It was held under the auspices of Oak Ridge Associated Universities from 18 to 20 April 1984, and sponsored by the National Institute of Diabetes, Digestive and Kidney Disease (NIDDK) and the National Cancer Institute (NCI), Oak Ridge Associated Universities (ORAU), and the National Foundation for Ileitis and Colitis. The planning committee members were as follows: Dr. W Brown (Colorado); Dr. N. Clapp (ORAU); Dr. D. Podolsky (Boston); Dr. R. Riddell (Chicago); Dr. A. Rogers (Cambridge, MA); Dr. K, Vener (NIDDK), and Dr. S. Wolman (NYU). The outcome was a special supplementary edition with 32 published articles [20,39] and only 4 other years have surpassed this number. Researchers who contributed were among the top in this area of endeavor and many are well known to this day [40]. Many previously published articles were incorporated into a volume on CTTs in 1993 with 30 titles, and where many of the previously published findings were recapitulated, so there is bound to be some duplication. However, there are published conference transcripts in the 1985 supplementary edition, and the same debates are ongoing today, originating from two discussion sessions and a concluding session. There were other supplementary special journal editions devoted to the CTT, such as that of 1994, published in Agents and Actions [1], arising from a workshop arranged by Dr. Neal Clapp at the World Congress Inflammation event in the Austria Center Vienna, 10–15 October 1993, hosted by the International Association of Inflammation Societies; and a 1996 conference edition in Alimentary Pharmacology and Therapeutics [40].

Out of the many discussions the principal author had with Dr. Neal Clapp, director of MARCOR (Marmoset Colony at Oak Ridge) and a primary mover of this entire edifice of research, one was especially of note. It was postulated that the reason liver metastasis had been absent was that the blood supply differed from that of humans. This appeared to partially emanate from Discussion 1, with a comment from a participant answering a question to the effect that he had said that the lesions resemble ischemic mucosal disease (page 33S of the supplement). In his reply, he said that he had not looked at blood flow, but at necropsy, which appeared to be normal. Dr. Clapp confirmed that the portal blood system in CTTs and humans were not dissimilar (personal communication), and that this did not explain the paucity of liver metastasis in CTTs. He and his colleagues suggested that this phenomenon was perhaps due to the inability of the cancer to invade the portal circulation for anatomic, immunologic, or physiologic reasons, in contrast to the ease with which it spreads to other distant organs and lymph nodes [41]. Over the years, the CEA/PSG (pregnancy specific β-1 glycoproteins) part of the Ig superfamily of proteins has included Callitrichid presentations at the 4th international workshop 22–25 August 1993 at Harvard Medical School in Boston; the 6th from 5 to 8 September 1995 at Hotel Este’rel, Montreal, QC, under the auspices of McGill University; the 13th from 11 to 14 August 2002 at The Millcroft Inn, Alton, Ontario, Canada, under the aegis of The University of Toronto, where CEA gene changes were implicated in the lack of liver metastasis; the 16th on 8–11 September 2019 at the Banff Centre for Arts and Creativity, Alberta, CA; and the 31st CEA/PSG Symposium, 3–4 October 2023 at @Ease, 1345 Avenue of the Americas, New York City, hosted by the Feinstein Institutes for Medical Research and the Cold Spring Harbor Laboratory.

It would be remiss of us to avoid mentioning the viral interactions with the CTT, given the many challenges we face today from viral vectors. The most common viruses affecting Callitrichidae, in common with other non-human primates, are coronaviruses [42,43]. Outbreaks of colitis and diarrhea at MARCOR implicated *Campylobacter fetus* subsp. *jejuni* in 20% and coronavirus-like particles in 24% of cases [44]. The latter organism has also been reported in monkeys [44].

### Limitations

In general, our studies and most others dealing with Callitrichidae species have small numbers. Specifically, tissue samples are collected at necropsy because of the endangered species provision, and the tissues are not fresh, adversely affecting the integrity of the sample.

## 5. Conclusions

We believe that our perspective has been fairly comprehensive, because we compared multiple antigens and used a wide repertoire of antibodies for an adequate comparison. We chose to be somewhat selective to keep the paper as concise as possible. Most articles in the literature emphasize habitat, social behavior, and other external threats such as zoonoses. Perspective articles are rare and our body of previously published CTT articles represents 1% of the entire bibliography on this remarkable animal.

## Figures and Tables

**Figure 3:**
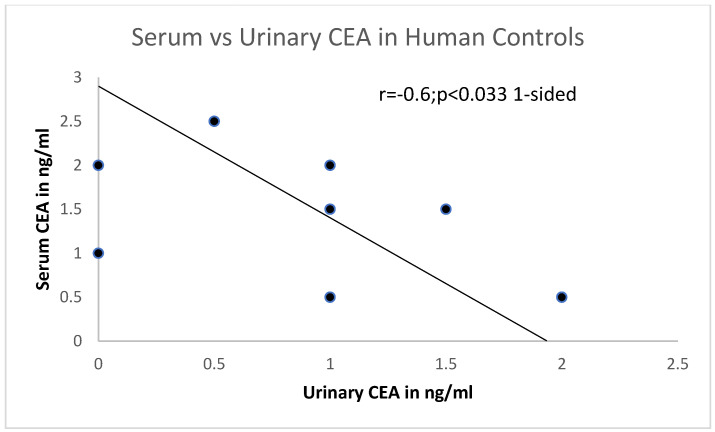
A putative inverse correlation is shown using the same assay between serum and urinary CEA from Egypt.

**Table 1:** Antigens and antibodies used.

Antigens	Type	Type	Designation	Use	Originator
CEA	ICAM; pro-met	IgG	T84.66; C1D2	Elisa, WB, IHC	J. Shively, C. Wagner
Mucin/CH_2_O	Le^a^ and Le^b^ *	IgM	SPan-1	IHC	Y.S. Kim
Fucosylated APG	Colorectal Cancer	IgM	CaCo 3/61	Elisa, IHC	A. Quaroni
Innate Immunity	Paneth cell prot	IgG2a	Adnab-9	Elisa, WB, IHC	M. Tobi
O.S. Neoantigen	Cancer and pre-Ca	IgG	Bac 18.1	Urine ELISA	Z. Bentwich et al.
Anti-k-*ras*	Tumor promotor	IgG	10a/AB-3	ELISA	NEN/OncogeneScience
Chromogranin A	Neuroendocrine	IgG	LK2HIO	ELISA, IHC	Hybritech
EGFR ^	Blood vessels	IgG	Ab1	ELISA	OncogeneScience

ICAM—intercellular adhesion molecule; pro-met—pro-metastasis; ELISA—enzyme-linked immunosorbent assay; WB—Western blotting; IHC—immunohistochemistry; IgG—immunoglobulin G; IgM—immunoglobulin M; CH_2_O—carbohydrate; Le—Lewis blood group; Prot—protein; * shares biological characteristics with CA19-9; ^ Paneth cell constituent, *p* < 0.005 CTT extracts; APG—aminoproteoglycans show highest expression in CTT extracts: *p* < 0.005—this *p* value portrays the differences between the CTT and humans; likewise O.S. neoantigen—organ specific neoantigen, specific to urinary colorectal neoantigens; Adnab-9 highest in normal control washings *p* < 0.05; and *ras*-p21 highest in human IBD human washings. An additional CEA assay was used (CEA AIA PACK kit (Tosoh Medics Inc., Foster City, CA, USA) because the 2 antibodies included were previously able to detect differences in CTT and CM CEA moieties [16]. The first is a solid phase Gold Class IV antibody and the second is Class 1 and is a more specific tracer, with a sensitivity limit of 0.05 ng/mL of CEA. The data were collected and stored in the Excel format on servers protected by a double firewall. Demographics of animals and humans were scored as well as outcomes of the above analytical tests. Telomerase assays were also performed by the University of Southwest Texas (Dr. J. Shay). This ribonucleoprotein contains a template of 11 nucleotides complementary to the (TTAGGG)*n* telomeric DNA repeats, as measured by the Trapeze assay (Oncor Inc., Gaithersburg, MD, USA). This can be used to detect tumors, but the activation usually occurs later in tumorigenesis.

**Table 2:** Percentage immunohistochemistry results in the tissues of CTT.

Antigen	CEA	SPAN-1	CaCo 3/61	Citation
Percent	0	20	100	[17]

No CEA staining was seen, as opposed to the reactivity of the other antibodies.

**Table 3:** CTT antigens reactive with CEA, mucins, and aminoproteoglycans.

Sample (n)	CEA ng/mL *	SPAN-1	CaCo 3/61
CTT Tissue Extract (9)	<1	0.010 ± 0.016	0.416 ± 0.236
CTT IBD Washing (11)	<1	0.091 ± 0.034	0.107 ± 0.056
IBD Human Washing (12)	664 ± 924	0.054 ± 0.059	0.261 ± 0.181
Normal Human Washing (14)	431 ± 449	0.150 ± 0.058	0.444 ± 0.658

Inflammatory bowel disease; * anti T84.66.

**Table 4:** Members of the CEA family expressed in CTT with and without cancer and controls.

ICAM Moiety (n)	CTT with Cancer (7)	Normal CTT (7)	CM Controls (7)
CEA ng/mL by CEA AIA *	778 ± 693 **	622 ± 357 **	104 ± 220
NCA 50/90 ^	0.60 ± 0.52	0.58 ± 0.35	1.55 ± 0.082
BGP ^+^ (CEACAM1)	0.294 ± 0.047	0.278 ± 0.031	0.278 ± 0.078

* Tosoh Medics Kit; ** *p* < 0.05 when compared to CM controls. ^ Non-specific cross-reacting antigen; ^+^ Biliary Glycoprotein.

**Table 5:** Putative mechanisms by which the CTT may evade liver metastasis.

Mechanisms	Molecule	% CTT	%CM	Statistics	Citation
1. PELPK-seq	CEACAM6	62	28.6	*p* < 0.043	[1,3]
2. BGP-staining	CEACAM1	0 in liver	0 in liver	*p* = 1	[2]
3. Glycosylation	CEACAM6	<2	<2	60% human	[27]
4. CEAR-staining	CEACAM6	42	nd	*p* < 0.05 vs. human	[3]
5. Fibulin-5 staining	Fibulin	60	40	NSS	[3]

Seq—sequencing; BGP—biliary glycoprotein; CEAR—carcinoembryonic antigen receptor; and NSS—not statistically significant.

## Data Availability

Data Availability Statements are available in the VHA directive 1200.12 of 3/9/2009. The VHA Handbook addresses both the use of data for research and the clinical and administrative data repositories for research. It also addresses the development and use of data research repositories.
